# A New Tissue Engineering Strategy to Promote Tendon–bone Healing: Regulation of Osteogenic and Chondrogenic Differentiation of Tendon‐derived Stem Cells

**DOI:** 10.1111/os.14152

**Published:** 2024-07-23

**Authors:** Sinuo Shen, Yucheng Lin, Jiachen Sun, Yuanhao Liu, Yuzhi Chen, Jun Lu

**Affiliations:** ^1^ School of Medicine Southeast University Nanjing China; ^2^ The Center of Joint and Sports Medicine, Orthopedics Department, Zhongda Hospital, School of Medicine Southeast University Nanjing China

**Keywords:** Cell differentiation, Signaling pathways, Sports medicine, Stem cells, Tissue engineering

## Abstract

In the field of sports medicine, repair surgery for anterior cruciate ligament (ACL) and rotator cuff (RC) injuries are remarkably common. Despite the availability of relatively effective treatment modalities, outcomes often fall short of expectations. This comprehensive review aims to thoroughly examine current strategies employed to promote tendon‐bone healing and analyze pertinent preclinical and clinical research. Amidst ongoing investigations, tendon‐derived stem cells (TDSCs), which have comparatively limited prior exploration, have garnered increasing attention in the context of tendon‐bone healing, emerging as a promising cell type for regenerative therapies. This review article delves into the potential of combining TDSCs with tissue engineering methods, with ACL reconstruction as the main focus. It comprehensively reviews relevant research on ACL and RC healing to address the issues of graft healing and bone tunnel integration. To optimize tendon‐bone healing outcomes, our emphasis lies in not only reconstructing the original microstructure of the tendon‐bone interface but also achieving proper bone tunnel integration, encompassing both cartilage and bone formation. In this endeavor, we thoroughly analyze the transcriptional and molecular regulatory variables governing TDSCs differentiation, incorporating a retrospective analysis utilizing single‐cell sequencing, with the aim of unearthing relevant signaling pathways and processes. By presenting a novel strategy rooted in TDSCs‐driven osteogenic and chondrogenic differentiation for tendon‐bone healing, this study paves the way for potential future research avenues and promising therapeutic applications. It is anticipated that the findings herein will contribute to advancing the field of tendon‐bone healing and foster the exploration of TDSCs as a viable option for regenerative therapies in the future.

## Graphical abstract

The graphical abstract outlines strategies for tendon‐bone healing using tendon‐derived stem cells (TDSCs). TDSCs, with high proliferation and differentiation capacities, are crucial for enthesis regeneration. Applications include injections, biomimetic systems, and physical therapy. Combining TDSCs with scaffolds and growth factors, guided by signaling pathways like TGF‐β, promotes healing.

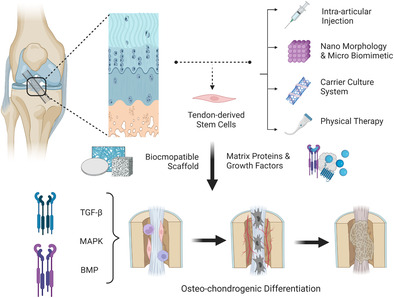

## Introduction

As sports gain increasing popularity, the number of tendon and ligament injuries has also risen with more than 40,000 cases reported annually worldwide.^1^, ^2^ Injuries to the anterior cruciate ligament (ACL) not only result in a decline in athletic performance but also raise the risk of osteoarthritis, imposing a huge health burden and disturbing daily living, negatively affecting physical and mental health.^3^, ^4^, ^5^, ^6^

**FIGURE 1:**
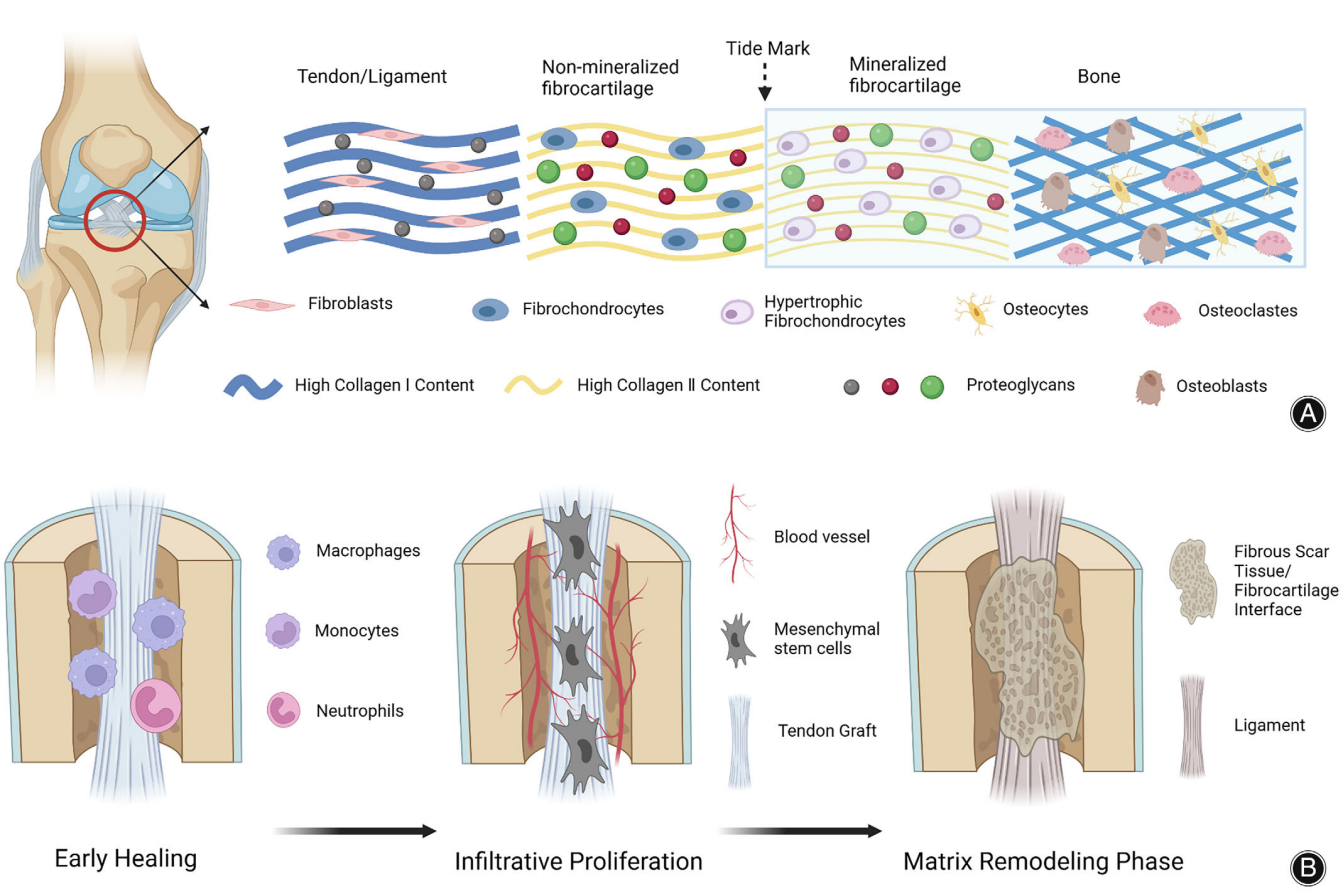
(A) Micro schematic representation of the tendon/ligament to bone insertion in the interface of the anterior cruciate ligaments. (B) Schematic diagram illustrating the graft healing process after ACL reconstruction. Created with BioRender.com.

The microstructure of the tendon–bone interface including ACL and rotator cuff (RC) are both direct insertion structures, which are composed of four parts, from tendon to bone, including ligaments, non‐mineralized fibrocartilage, mineralized cartilage, and bone (Figure 1A).^2^, ^7^ ACL injuries often instigated by sudden knee trauma, paralleling acute trauma models used in animal experiments. This alignment ensures more relevant findings. Shifting to the rotator cuff, disruptions at the tendon‐bone interface within the rotator cuff result from chronic overuse, trauma, or gradual degeneration. These injuries significantly impact shoulder mobility and frequently require intervention. So during the surgical reconstruction process, the surgeon usually uses various tendon grafts, sutures, screws, plates, and other methods to fix it on the bone tunnels. This procedure includes stages like removing damaged ligaments, graft preparation, drilling of bone tunnels, and graft fixation.^8^

During the healing process, the tendon–bone interface undergoes a number of stages, including the invasion by early inflammatory cells, the development of fibrous vascular tissue, and the proliferation and differentiation of late stem cells.^9^, ^10^ Tendon grafts must be remolded in ultrastructure to complete the function of ligament and bone to transmit force. However, following anterior cruciate ligament reconstruction (ACLR), the original form of the typical tendon–bone insertion interface is difficult to recover. Poor restoration of mechanical properties and original structures is a major challenge.^7^, ^11^, ^12^ Excessive inflammatory reaction, poor mechanical stress and relative degradation, gradient structure irregularity, and even the creation of scar fiber structure are frequent causes for the bad result of reconstruction surgery.^13^, ^14^, ^15^ Therefore, many related topics have been derived around this series of issues, including the elimination of inflammatory reactions, the promotion of angiogenesis, the use of cell‐free extracellular matrix (ECM) materials, and the use of stem cell secretions.^16^, ^17^ Among the options, one of the promising strategies is stem cell‐based therapy, because it has the ability to self‐renew and induce differentiation and can stimulate tissue regeneration through exogenous or endogenous regulatory mechanisms.^18^, ^19^

Among the various types of stem cells, bone marrow mesenchymal stem cells (BMSCs) are the most important because of their potential in tendon–bone healing. BMSCs have been demonstrated to have the ability to differentiate into multiple cell types, including osteoblasts, chondrocytes, and tendon cells, and release growth factors and cytokines that encourage tissue regeneration.^9^, ^20^ However, there is considerable interest in the possibility of additional types of stem cells for tendon–bone repair, such as tendon‐derived stem cells (TDSCs). TDSCs can be easily obtained from tendon tissue and have shown greater differentiation potential than BMSCs.^21^, ^22^

Several studies have demonstrated the promise of TDSCs in facilitating tendon–bone repair. For example, a study by He *et al*. investigated the potential of TDSCs in enhancing the healing of RC tendon–bone interface.^23^ Another study by Lui *et al*. demonstrated that TDSCs can enhance the healing of the tendon–bone interface, encourage fibrocartilage production, and facilitate tunnel bone integration, which is essential for the mechanical stability of the transplant.^24^ Although the results are promising, there are still several challenges to be solved before TDSCs can be used in clinical applications, such as safety concerns, limited availability, inconsistent differentiation, the tendon microenvironment, and delivery methods.

To address the problem of restoring the tendon–bone healing following ACL surgery, a unique research paradigm is required. This approach should aim to effectively stimulate the migration, proliferation, and differentiation of TDSCs, ultimately promoting the healing of the tendon–bone interface. We followed the methods of Font Tellado *et al*., and this review methodically reviews the development of tendon‐bone healing based on the reconstruction of the anterior cruciate ligament. It also explores the application strategies of TDSCs and their potential value. In addition, this work provides a thorough description of the transcriptional and molecular regulatory processes influencing the differentiation of TDSCs.^19^

We conducted a systematic literature search using PubMed, Google Scholar, and Web of Science, focusing on publications from the past two decades. We used a combination of specific and broad keywords including “tendon‐to‐bone healing,” “tendon‐bone interface,” “biomaterials,” “stem cells,” “tissue engineering scaffolds,” “regenerative medicine,” “orthopedic” “signal pathway,” “osteogenesis,” “chondrogenesis,” and “tendon‐derived stem cells.” Boolean operators such as “AND,” “OR,” and “NOT” were employed to refine the search results. Studies were selected based on relevance, methodological rigor, and significance to the field, ensuring a comprehensive overview of current research in tendon‐to‐bone healing.

Inclusion criteria were: (i) studies published in peer‐reviewed journals; (ii) research articles, systematic reviews, and clinical trials focused on tendon‐to‐bone healing; (iii) studies published within the last two decades; (iv) articles written in English; and (v) research involving biomaterials, stem cells (tendon‐derived stem cells), biomechanics, tissue engineering scaffolds, and regenerative medicine related to tendon‐bone repair.

## Mechanism of Tendon–bone Healing

Next, the investigation will concentrate on elucidating the cellular and molecular mechanisms underlying tendon–bone healing at the tendon–bone interface subsequent to ACL reconstruction. Initially, the avascular gradient at the tendon–bone interface triggers an inflammatory response, attracting macrophages and mesenchymal stem cells (MSCs). Macrophages clear debris and activate MSCs.^25^, ^26^ MSCs, recruited from the bone marrow and synovium, differentiate into tenocytes and osteoblasts. Tenocytes produce collagen, while osteoblasts induce new bone formation, both essential for tendon–bone repair.^9^, ^27^, ^28^

Early post‐surgery, the bone‐ligament interface consists mainly of fibrous tissue. The modifications in the bone tunnel wall resemble the process of endochondral ossification, and the bone tunnel environment mimics a fracture. Osseointegration, the growth of bone into the graft, plays a critical role in tendon–bone healing.^25^ Over time, this tissue organizes into a more structured form. Studies show fibroblasts appearing at 4 weeks, and Sharpey's fibers forming at 6–8 weeks, strengthening the attachment (Figure 1B).^29^, ^30^, ^31^ Vascularization occurs progressively throughout time, facilitating the metabolic needs of the repairing tendon–bone junction.^2^, ^31^ The distal end of fibrocartilage tendon was formed by layering of tendon, fibrocartilage, calcified cartilage, and bone structures, which occur 12 weeks after reconstruction. It is crucial to note that the indirect stop point is merely a temporary circumstance, as validated by Kuang *et al*.^32^ This complex cellular response and matrix remodeling ultimately lead to the establishment of a fibrocartilage zone between the tendon and bone which provides mechanical strength at the interface.^33^

On the other hand, tendon graft integration involves new blood vessel development, collagen synthesis, and remodeling to mimic natural ligament structure and function. Achieving this process is vital for long‐term joint stability.^26^, ^34^, ^35^ Comprehending this process is imperative for achieving long‐term joint stability and functionality. Tendons, mainly composed of parallel collagen bundles, differ from ligaments, which have a more integrated collagen fiber arrangement and higher fibroblast density.^36^ Different treatment strategies have been developed based on the above content.

## Biomaterials for Tendon‐bone Healing

After ACL reconstruction, conservative treatment typically involves physical therapy and rehabilitation to enhance knee stability and function (Table 1).^42^

**TABLE 1:** Physical treatment in tendon‐bone healing.

Intervention/treatment	Results	Study design/animal model	References	Cells/differentiation
LIPUS	May aid in the initial phase of tendon‐bone healing process in patients who have undergone rotator cuff repair. This treatment may also be beneficial following other types of reconstructive surgeries involving the tendon‐bone interface.	Extra‐articular transosseous‐equivalent ovine rotator cuff model	37	MSC osteogenic differentiation
LIPUS	LIPUS treated group showed a higher bone volume/total volume ratio and better TBI maturity score, with a significantly higher failure load than the control group, promoting anti‐inflammatory macrophage polarization (M2) in the late phase.	Rat model of rotator cuff tear	38	Macrophage polarization
ESWT	ESWT had similar effects between the low and high dose for treating delayed TBI healing.	Rabbit partial patellectomy model	39	Not mentioned
Electrical stimulation	ES has a positive osteogenic effect at the cellular and molecular levels, providing interesting clues for the potential mechanism of promoting bone healing.	*In vitro* experiments	27	MSC osteogenic differentiation
Mechanical stress load	Immobilization results in a stronger tendon‐bone complex, with less scar tissue and a more organized tendon‐bone interface compared to all loading regimens in this study.	Rat model of unilateral patellar tendon detachment	40	Not mentioned
PEMF	MED‐generated PEMF may enhance early postoperative tendon‐to‐bone healing, superior biomechanical elasticity parameters together with better collagen organization suggest improved RC healing.	Acute bilateral supraspinatus tear and repair model in Wistar rats	41	Not mentioned

ESWT, extracorporeal shockwave therapy; LIPUS, low‐intensity pulsed ultrasound; MED, miniaturized electromagnetic device; MSC, mesenchymal stem cell; PEMF, pulsed electromagnetic field; RC, rotator cuff; TBI, tendon‐bone interface.

Various physical treatments, such as low intensity pulsed ultrasound (LIPUS), extracorporeal shockwave therapy (ESWT), electrical stimulation (ES), mechanical stress load, and pulsed electromagnetic field (PEMF), have been evaluated for their effectiveness in promoting tendon‐bone healing. Further research is needed to optimize treatment protocols and validate their clinical effectiveness (Table 1).

Initial biomaterials like stainless steel had biocompatibility and corrosion issues, leading to the development of advanced alloys like titanium (Ti) and its alloys. Ti alloys, enhanced with tantalum (Ta) and surface modifications, improve osseointegration.^43^ Ceramic biomaterials, such as hydroxyapatite/type I collagen (HAp/Col I), show promise in ACL healing by promoting cell growth and osteogenic expression.^44^, ^45^

Current research focuses on optimizing biomaterials for tendon‐bone healing by exploring surface modifications, biodegradable plans, and advanced fabrication methods.^46^, ^47^, ^48^ Nanotechnology advancements have led to the development of sophisticated composite scaffolds with tailored properties for tissue engineering. For instance, nanoscale ZIF‐8 enhances bone regeneration by promoting stem cell differentiation, while nano‐collagen offers superior mechanical stability.^49^, ^50^ Natural fish scale modified by calcite nanoparticles (CS‐FS) demonstrates excellent strength and bioactivity, making it a promising biomaterial for clinical use.^51^

Tissue engineering aims to repair and regenerate tendons and bones by controlling the healing microenvironment. This involves adjusting scaffold materials and delivering signaling molecules to promote appropriate cell differentiation and tissue formation.^2^, ^52^, ^53^ Scaffolds can offer a framework for cell attachment and growth and convey growth factors and other signaling molecules to the wounded location, such as hydrogels, has also been shown to promote osteo‐chondrogenic differentiation and enhance the therapeutic potential of TDSCs in tendon–bone healing (Table 2).^62^, ^64^, ^66^, ^72^, ^73^

**TABLE 2:** Biomaterials in tendon‐bone healing.

Intervention/treatment	Results	Study design/animal model	References	Cells/differentiation
Mg‐Zn‐Sr alloy screws	Provided sufficient mechanical strength to fix the tendon graft during the entire graft healing period and effectively attenuated peri‐tunnel bone loss as potential fixators in ACL reconstruction.	Rabbit model of ACL reconstruction	54	Not mentioned
Zn‐Mn‐Mg alloy screws	Accelerated the formation of new bone and induced partial tendon mineralization, promoting tendon‐bone integration.	ACLR model of rabbit	55	hBMSC osteogenic differentiation
Mg screws	Accelerated and significantly increased mineralized matrix formation at the tendon‐bone interface in animals compared to those with Ti screws.	Rabbit model of ACL reconstruction	56	BMSCs osteogenic differentiation
Mg screws	Accelerated fibrocartilaginous entheses regeneration in ACL reconstruction, potentially due to the stimulation of BMP‐2 and VEGF by Mg ions.	Rabbit model of ACL reconstruction	57	Fibrocartilage differentiation
OCP	Promoted osteogenic differentiation of TSPCs, related to changes in ionic concentration and pH in the chemical environment caused by OCP hydrolysis.	*In vitro* experiment	58	TSPC osteogenic differentiation
ICPCB composite	Had a porous structure and better osteointegration effect, with direct clinical instruction to arthroscopic techniques of ACL reconstruction.	Bilateral ACLR model in rabbits	59	Osseointegration
Hap/PLGA scaffold	Fabricated a hierarchical structured scaffold promising for the repair of tendon to bone insertion, with seeded stem cells exhibiting directed differentiation into tenocytes and osteoblasts along the mineral gradient as a response to the graded Young's modulus.	*In vitro* experiment	60	ASC tendon differentiation/osteogenic differentiation
Bipolar metal flexible electro‐spun fibrous membrane	Verified tendon‐bone interface integration regeneration ability through *in vitro* and *in vivo* experiments with gradient generating ability.	Rat rotator cuff tear model	61	*In vitro* BMSC osteogenic differentiation
PCL/COL‐1 hybrid electro‐spun nanofiber membranes	Supported cell adhesion, proliferation, and osteogenic differentiation of TSPCs, acting as a suitable functional biomimetic scaffold in tendon‐bone tissue engineering applications to enhance tendon‐bone healing abilities.	*In vitro* experiment	62	TSPC osteogenic differentiation
Triple network hydrogel	Developed a strong, hard, adhesive hydrogel mimicking osteo‐adherent tendon, achieving strong osteo‐adhesiveness similar to that of natural ligaments.	*In vitro* simulation experiments	63	Not mentioned
Cu and Zn ion bimetallic ion hydrogels	Promoted regeneration of tendon and bone simultaneously, reconstructing the microstructure of the tendon‐bone insert with antibacterial and regenerative abilities.	Rat rotator cuff tear model	64	*In vitro* osteogenic differentiation of osteoblasts
Composite hydrogel	Mediated sustained *in situ* release of curcumin and Mg2+ to effectively promote rotator cuff tendon‐to‐bone healing *via* anti‐inflammatory and pro‐differentiation effects.	Rat rotator cuff tear model	65	MSC chondrogenic differentiation
Gradient hydrogel scaffolds	Systematically screened for osteoblast differentiation, with the mechanical characteristics of the matrix impacting the 3D differentiation of osteoblasts.	*In vitro* experiment	66	Osteogenic differentiation and mineralization in MC3T3‐E1 cell lines
Kartogenin (KGN), a biocompound	A small molecule called KGN may be a promising approach to enhance and accelerate the formation of cartilage‐like tissue in the TBJ tendon/bone interface as well as ACL reconstruction.	*In vitro* experiment/rat Achilles tendon injury model	67	BMSC/TSPC chondrogenesis
3D collagen‐glycosaminoglycan (CG) scaffold	Selective structural modification to a 3D CG scaffold combined with biochemical supplementation can drive human bone‐marrow‐derived MSC differentiation down tenogenic, osteogenic, and chondrogenic lineages.	*In vitro* experiment	68	BMSC myogenesis, osteogenesis, chondrogenesis
Layer‐specific, GFs‐loaded μS	A living tissue construct with layer‐specific, GFs‐loaded μS can direct *in situ* and region‐specific differentiation of the embedded stem cells to tenogenic, chondrogenic, and osteogenic lineages for functional regeneration of the enthesis tissues.	Rat rotator cuff tear model	69	hUCMSC myogenesis, osteogenesis, chondrogenesis
GF‐embedded 3D‐printed scaffolds	Findings demonstrate the potential of *in situ* tissue engineering of tendon‐to‐bone interfaces by endogenous progenitor cells.	Rat rotator cuff tear model	52	TSPCs
Functional PPC sheets wrapping the graft	Periosteal progenitor cell monolayer maintains the differentiated capacity and osteochondral potential to promote fibrocartilage formation in tendon‐bone junction.	Rabbit ACL reconstruction model	70	Not mentioned
Mg‐pretreated periosteum wrapping the graft	Mg‐pretreated periosteum (M‐P) significantly enhanced the osteointegration of the tendon graft into the bone tunnels in rabbits as compared to the SS‐P group.	Rat ACL reconstruction model	71	PDSC osteogenesis
Hydrogel encapsulating curcumin and Mg	The composite hydrogel mediated sustained *in situ* release of curcumin and Mg2+ to effectively promote rotator cuff tendon‐to‐bone healing *via* anti‐inflammatory and pro‐differentiation effects.	Rat rotator cuff tear model	65	Stem cell recruitment and chondrogenesis

ACL, anterior cruciate ligament; ASC, adipose‐derived stem cell; BMP‐2, bone morphogenetic protein 2; BMSCs, bone marrow mesenchymal stem cells; CG, collagen‐glycosaminoglycan; COL‐1, collagen type I; GFs, growth factors; Hap, hydroxyapatite; hBMSC, human bone marrow mesenchymal stem cell; hUCMSC, human umbilical cord mesenchymal stem cell. GF‐embedded 3D‐printed scaffolds, growth factor‐embedded three‐dimensional printed scaffolds; ICPCB, *in‐situ* calcium phosphate ceramic–biodegradable polymer; MC3T3‐E1, mouse calvaria 3T3 cell line; Mg, magnesium; Mg‐Zn‐Sr alloy, magnesium‐zinc‐strontium alloy; M‐P, magnesium‐pretreated periosteum; MSC, mesenchymal stem cell; OCP, octacalcium phosphate; PCL, polycaprolactone; PDSC, periosteal‐derived stem cell; PLGA, poly (lactic‐co‐glycolic acid); PPC, periosteal progenitor cell; SS‐P, stainless steel‐pretreated periosteum; TBJ, tendon‐bone junction; Ti, titanium; TSPCs, tendon stem/progenitor cells; VEGF, vascular endothelial growth factor; Zn‐Mn‐Mg alloy, zinc‐manganese‐magnesium alloy; μS, microspheres.

Various biomaterials, including alloy screws, scaffolds, hydrogels, and small molecules, have been studied for their potential to enhance tendon‐bone healing. These materials promote osseointegration, osteogenic differentiation, and tissue regeneration at the tendon‐bone interface, underscoring the importance of innovative biomaterial‐based approaches in improving outcomes for procedures like ACL reconstruction and rotator cuff repair (Table 2).

## Seed Cells in Tendon‐bone Healing

### Cell Types Mainly Involved in Tendon–bone Healing Interface

The healing process at the tendon–bone interface in the bone tunnel after ACL reconstruction involves several cell types and biological processes, including mesenchymal stem cells (MSCs), adipose‐derived stem cells (ASCs), TDSCs, synovium‐derived MSCs, periosteal stem cells, vascular endothelial cells, and smooth muscle cells.^9^, ^14^, ^25^ Among these cells, MSCs are capable of self‐renewal and differentiation into osteoblasts, chondrocytes, and fibroblasts, contributing to new tissue development. ASCs have been shown to promote tissue regeneration in tendon–bone healing due to their ability to secrete paracrine factors that stimulate angiogenesis, proliferation, and differentiation of MSCs.^74^ Tendon stem/progenitor cells and synovium‐derived MSCs proliferate and differentiate on scaffolds and recruit endogenous tendon stem/progenitor cells, leading to improved integration of the tendon–bone interface.^75^, ^76^ Another study by Jones and Pei investigated the potential of synovium‐derived MSCs for promoting regeneration of the cartilage in a rabbit model.^77^ Periosteal stem cells, derived from the periosteum, can secrete growth factors and extracellular matrix on the scaffold and participate in the mineralization process in the bone tunnel.^70^ These diverse stem/progenitor cells and vascular cells collaborate to regenerate multilayer structures such as fibro‐synthetic tissue, fibro‐synthetic cartilage, mineralized cartilage, and bone tissue during tendon–bone repair.^78^

### Endogenous and Exogenous Stem Cells

**FIGURE 2:**
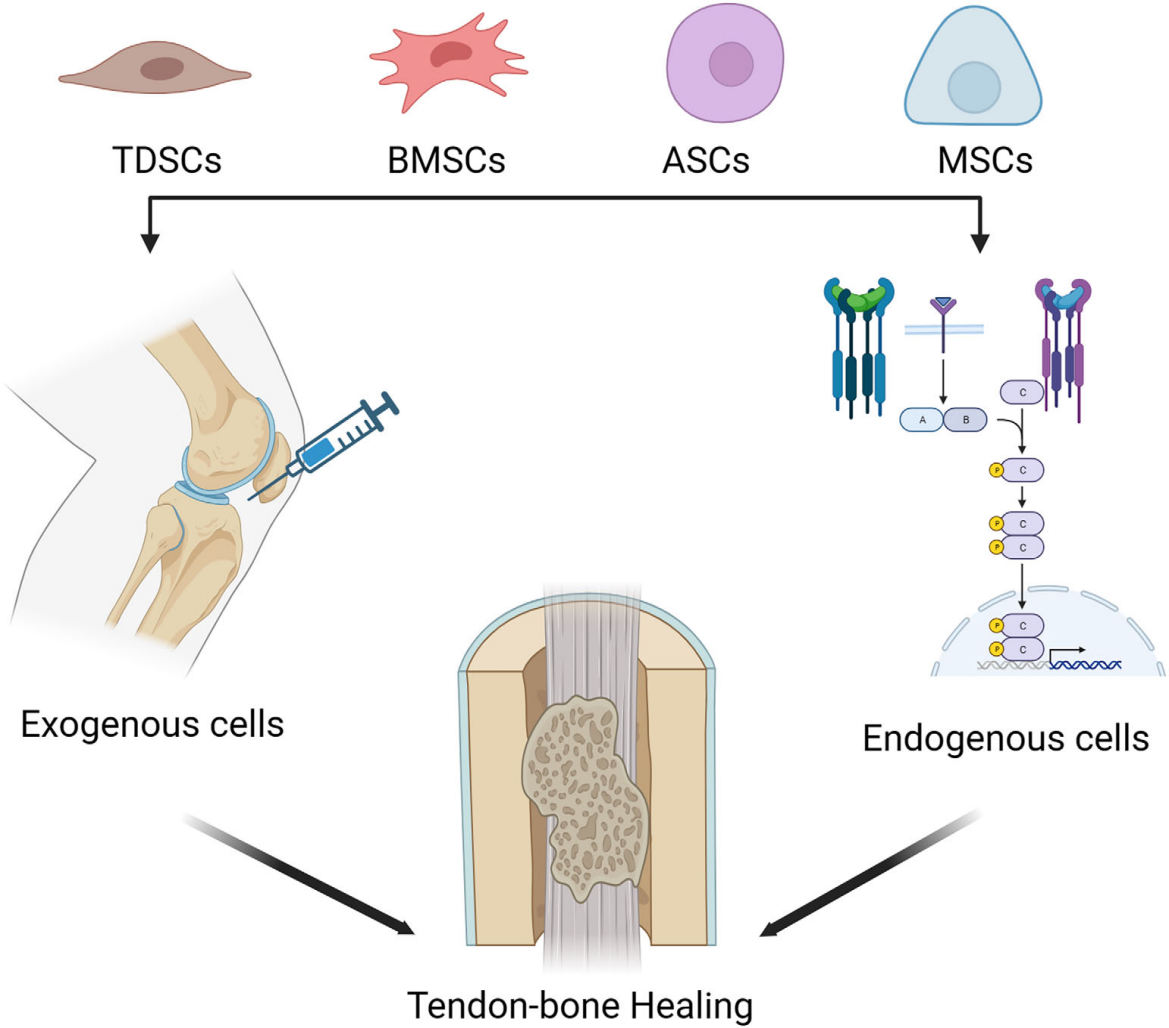
Schematic diagram of endogenous and exogenous stem cells involved in tendon‐bone healing. Created with BioRender.com.

During the course of research, it has been discovered that stem cells are essential for tendon–bone repair. Endogenous stem cells, which originate from a patient's own tissue, can be induced to differentiate into the desired cell types, thereby reducing the risk of rejection and other complications associated with the use of exogenous cells.^79^ This is a promising strategy for promoting tendon–bone healing (Figure 2).^52^ This method utilizes the patient's own cells, minimizing the risks of rejection and other complications associated with the transplantation of exogenous cells. Consequently, it is crucial to comprehend the many signaling networks that control stem cell differentiation and create precise techniques to alter these pathways for best results.^80^ Endogenous stem cells are naturally scarce and quiescent in adult tissues, posing additional challenges for their successful induction of differentiation.^81^, ^82^

On the other hand, exogenous stem cells obtained from a donor or a patient's own tissue can provide a large number of cells that can be differentiated into the desired cell types.^78^, ^83^, ^84^ This method entails getting stem cells from outside sources, including donors or the patient's own tissue, and putting them into the wounded location.^85^, ^86^ However, several key factors, including the source, delivery method, and optimal dosage of stem cells, must be considered when implementing this method. Further study should concentrate on enhancing the use of stem cells in clinical settings. Strategies such as gene editing and tissue engineering are being explored to enhance the survival and integration of transplanted stem cells. Novel delivery systems, including hydrogels and nanoparticles, are being developed to enhance procedure efficacy and safety.^63^, ^64^, ^65^

Integrating cell differentiation with exogenous stem cell supplementation can provide a synergistic effect for promoting tendon–bone healing. For instance, the use of exogenous stem cells can offer a larger cell bank to integrate into the injured area, while these stem cells can be activated and directed toward specific cell types needed for recovery.^2^, ^9^, ^68^, ^87^ In a study by Lui *et al*., the researchers showed that TDSCs, when formed into cell sheet and implanted into a rabbit model of ACL reconstruction, dramatically enhanced tendon–bone healing compared to a control group.^24^ Similarly, Zhao *et al*. reported that the application of TDSCs led to improved tendon–bone healing and biomechanical properties in a rat model of ACL reconstruction.^88^

### Tendon‐derived Stem Cells

**FIGURE 3:**
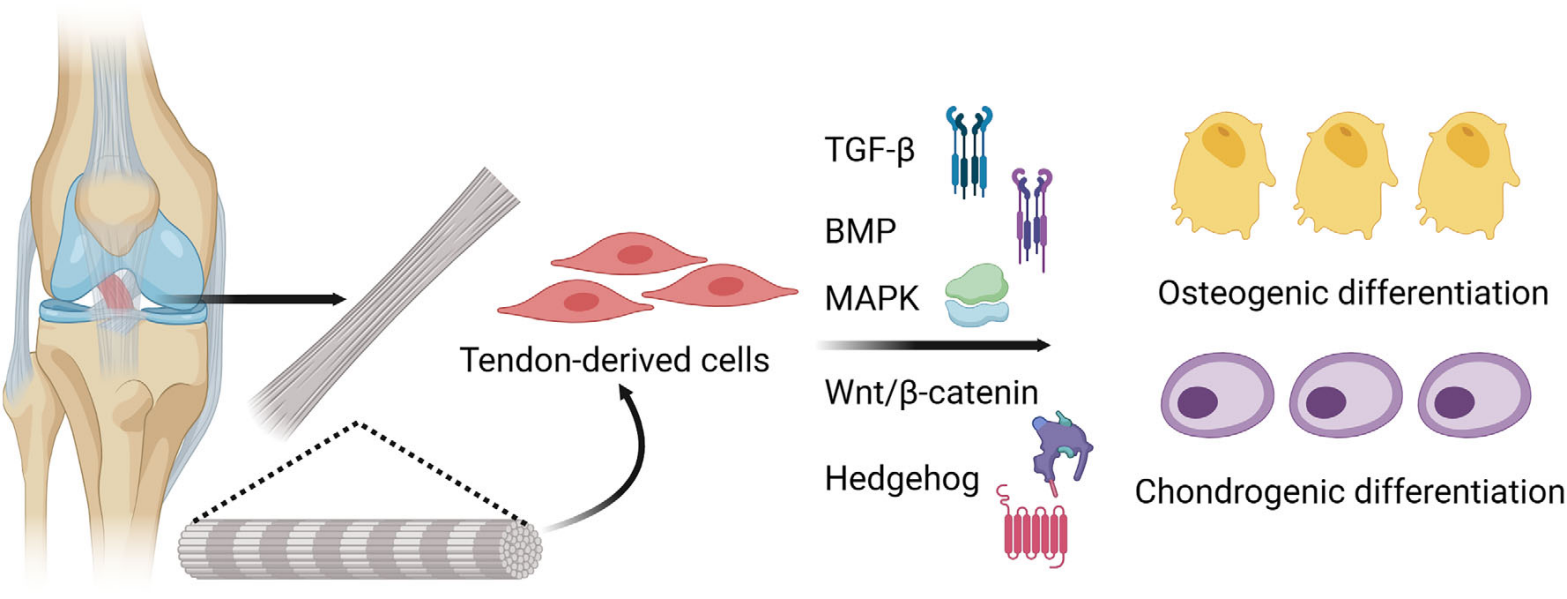
The multifunctionality of tendon‐derived stem cells and related cytokines and mechanisms. Created with BioRender.com.

During embryonic development, the mesoderm constitutes one of the three basic germ layers and gives rise to various tissues, including tendons. The mesoderm differentiates into multiple lineages, such as somites that act as precursors to the vertebrate skeleton. Sclerotome cells arise from the somites, forming the spine, and produce precursors for tendons, ligaments, and intervertebral discs.^21^, ^76^, ^81^ As development proceeds, these tendon precursors differentiate into TDSCs and initiate the production of the ECM that composes the tendon. While many signaling pathways and transcription factors, including transforming growth factor‐beta (TGF‐β), bone morphogenetic protein (BMP), and tenomodulin, control ECM formation, certain crucial aspects of TDSCs like their precise niche inside tendons or surface protein expression profiles remain unclear.^88^ TDSCs have shown potential in promoting tendon–bone healing, which is critical after ligament reconstruction procedures (Figure 3). Their multipotency allows them to differentiate into tenocytes, osteoblasts, adipocytes, and chondrocytes.^21^, ^62^, ^80^, ^82^

TDSCs have the ability to differentiate into osteoblasts, the cells responsible for bone formation, which is vital for successful tendon–bone healing and create bone‐specific extracellular matrix proteins. They promote the osseointegration process and increase the biomechanical characteristics of the healing interface. A study conducted on rat TDSCs revealed their capacity to undergo osteogenic differentiation when exposed to 2% elongation uniaxial mechanical tension for 3 days. This was proven by the increased expression of important indicators such as Runt‐related transcription factor 2 (Runx2), collagen type 1 α1 (Col1α1), alkaline phosphatase (ALP) activity, and ALP cytochemical staining.^89^

According to the literature, the bone tunnel remodeling shares similarities with the process of endochondral ossification, and the environment of the tunnel resembles that of a fracture.^2^, ^67^, ^70^, ^90^ Bone ingrowth is critical for successful graft‐to‐bone healing, as it coincides with the improvement from loading to failure. Numerous studies have investigated ways to enhance bone ingrowth into tendon transplants.^91^ Thus, promoting the differentiation of TDSCs toward the cartilage and bone is crucial for successful tendon–bone healing. An effective healing environment necessitates differentiation in osteo‐chondrogenic directions.^92^

By imitating the natural ligament's structure and function and encouraging fibrocartilage production, TDSCs contribute to better osteogenesis and bone integration around the graft.^24^, ^25^, ^88^ Chondrocytes contribute to the formation of fibrocartilage, which is vital for successful tendon–bone healing. Fibrocartilage acts as a bridging tissue between the tendon and bone, promoting correct integration and biomechanical performance.^75^, ^91^, ^93^ Research has found that tendon progenitor cells have stronger chondrogenic potential under pathological conditions compared to progenitor cells in uninjured normal tendons.^75^ TDSCs were successfully stimulated to develop into a fibro‐chondrogenic lineage *in vitro* using transforming growth factor‐beta 3 (TGF‐β3) and BMP2. Following transplantation into immunocompromised nude mice after seeding in a collagen II sponge, the TDSCs maintained their fibro‐chondrogenic characteristics, as evidenced by the expression of proteoglycan, collagen I, and collagen II.^93^ This strategy may allow for the creation of techniques to fix specific cartilage abnormalities and possibly stop the progression of osteoarthritis.^94^ Adipogenic differentiation is not typically required in the context of tendon–bone healing, as it may interfere with proper tissue integration, especially given studies indicating that aging makes TDSCs more prone to adipogenic differentiation.^95^

But there are still several unresolved concerns that need to be addressed before TDSCs can be applied in clinical settings. One issue is the heterogeneity of TDSCs, which can vary depending on the source of the cells and the culture conditions used. This may hinder the capacity of TDSCs to develop into tenocytes and osteoblasts and promote tissue regeneration. Another issue is the potential for TDSCs to form fibrous tissue or scar tissue instead of functional tendon and bone tissue.^58^ This is a typical concern in tissue engineering, as the newly created tissue frequently lacks the functional qualities of the native tissue. Therefore, strategies need to be developed to promote the formation of functional tendon and bone tissue, such as the use of growth factors or the development of biomimetic scaffolds that mimic the structure and composition of native tissue.

## Cytokine and Mechanism in the Study of Tendon‐derived Stem Cells

One of the key factors in inducing osteo‐chondrogenic differentiation is the selection of appropriate growth factors and cytokines. Studies have reported that TGF‐β1, BMP2, and insulin‐like growth factor 1 (IGF‐1) can stimulate the osteo‐chondrogenic development of TDSCs, both *in vitro* and *in vivo*.^22^ The efficacy of such intervention hinges on the intricate interplay between TDSCs and biomaterials, orchestrated through complex signaling pathways governing osteogenesis and cartilage differentiation. A range of biomaterials has been under scrutiny, bolstering TDSCs‐mediated tissue repair with plausible connections to TGF‐β, BMP, Mitogen‐activated protein kinase (MAPK), wingless/integrated/β‐catenin (Wnt/β‐catenin), and mHedgehog pathways (Figure 3). This interaction significantly influences TDSC‐driven osteogenesis and cartilage formation. These signaling pathways intricately intersect with TDSCs and biomaterials, ultimately shaping the outcomes of tissue engineering strategies (Table 3).^19^

**TABLE 3:** Biomaterials loaded with growth factors or cells in tendon‐bone healing.

Intervention/'treatment	Results	Study design/animal model	References	Cells/differentiation
Porous membrane with reverse gradients of PDGF‐BB and BMP‐2	The membrane sections with higher PDGF‐BB and lower BMP‐2 concentrations provided a better environment for ASC tenogenesis, while the membrane sections with higher BMP‐2 and lower PDGF‐BB concentrations were better for promoting osteogenesis.	*In vitro* experiment	96	*In vitro* ASC tenogenesis and osteogenesis
MEG3	Studies reported the positive and negative osteogenic effects of MEG3 in MSCs.	*In vitro* experiment	97	MSC osteogenesis
Encapsulation of decellularized tendon scaffolds with CDSC or BMSC gels	CDSCs exhibit higher self‐renewal capacities, better adaptability to low‐oxygen and low‐nutrient post‐transplantation environments, as well as strong bi‐potent differentiation abilities of osteogenesis and tenogenesis.	Rat patellar tendon injury model	98	BMSCs/CDSCs osteogenesis and tenogenesis
Exosomes from IPFP MSCs	IPFP MSC‐derived exosomes accelerated tendon‐bone healing and intra‐articular graft remodeling after ACLR, which may have resulted from the immunomodulation of macrophage polarization.	Rat ACL reconstruction model	99	M1 macrophage to M2 macrophage polarization
BMSC‐Exos	BMSC‐Exos promoted M1 macrophage to M2 macrophage polarization *via* miR‐23a‐3p, reduced the early inflammatory reaction at the tendon‐bone interface, and promoted early healing after ACLR.	Rat ACL reconstruction model	9	M1 macrophage to M2 macrophage polarization
PRP (with carrier) simvastatin‐containing PRP gel	The combination of simvastatin with PRP induced chondrogenesis of BMSCs *in vitro* and enhanced fibrocartilage formation *in vivo*. The simvastatin‐PRP gel treatment promotes wounded tendon‐bone interface healing in clinical treatment.	Rat Achilles tendon injury model	100	BMSC chondrogenesis
PRP‐GelMA hydrogel	The PRP in the construct could promote tissue repair significantly by facilitating the proliferation and differentiation of MSCs. This composite can create a reparative environment through M2 polarization effectively for osteochondral regeneration.	Rabbit osteochondral defect model	72	BMSC osteogenesis and chondrogenesis
3D print of miR transfected ADCs spheroids	miR‐transfected ADSC spheroids have osteogenic and chondrogenic potential for use at the tendinous bone healing interface.	*In vitro* experiment	101	ADSC chondrogenesis and osteogenesis
Scx‐overexpressing PDGFRα(+) BMMSCs	BMMSC‐Scx‐exos or miR‐6924‐5p could serve as a potential therapy for the treatment of osteolysis during tendon‐bone healing and improve the outcome.	Mouse tendon‐bone tunnel model	102	Inhibit peritunnel osteolysis
Complex between LK peptides and miRNA 2861	cLK effectively delivered miRNA 2861 into the cytoplasm of human MSCs and accelerated osteogenic differentiation from MSCs, as well as mineralization.	*In vitro* experiment	103	MSC osteogenesis
ADSC with miR‐150‐5p inhibition laden in hydroxyapatite/tricalcium phosphate ceramic powders	The combination of ADSCs with miR‐150‐5p inhibition and hydroxyapatite/tricalcium phosphate ceramic powders enhanced bone regeneration.	Murine subcutaneous implantation model	104	ADSC osteogenesis
lncRNA MALAT1/gene transfection	MALAT1 promoted osteogenic differentiation and inhibited cell apoptosis through the miR‐485‐5p/WNT7B axis, which suggested that MALAT1 is a potential target to alleviate osteoporosis.	Hindlimb unloading mice model	105	Pre‐osteoblast cell

3D, three‐dimensional; ADSCs, adipose‐derived stem cells; ASC, adipose‐derived stem cell; BMP‐2, bone morphogenetic protein‐2; BMSC, bone marrow‐derived mesenchymal stem cell; BMSC‐Exos, bone marrow mesenchymal stem cell‐derived exosomes; CDSC, chondrogenic‐differentiated stem cell; Exos, exosomes; GelMA, gelatin methacrylate; IPFP MSCs, infrapatellar fat pad mesenchymal stem cells; LK peptides, leucine‐lysine peptides; lncRNA, long non‐coding RNA; M1 macrophage, pro‐inflammatory macrophages; M2 macrophage, anti‐inflammatory/pro‐regenerative macrophages; MALAT1, metastasis associated lung adenocarcinoma transcript 1; MEG3, maternally expressed gene 3; miR, microRNA; miR‐485‐5p, microRNA 485‐5prime; miRNA, microRNA; MSC, mesenchymal stem cell; PDGF‐BB, platelet‐derived growth factor‐BB; PDGFRα(+) BMMSCs, platelet‐derived growth factor receptor alpha‐positive bone marrow mesenchymal stem cells; PRP, platelet‐rich plasma; Scx, scleraxis; WNT7B, wingless‐type MMTV integration site family, member 7B.

Various biomaterials loaded with growth factors or cells have shown promising results in promoting tendon‐bone healing. These interventions include porous membranes with gradients of growth factors, such as PDGF‐BB and BMP‐2, which provide environments conducive to tenogenesis and osteogenesis. Additionally, studies have explored the effects of molecules like MEG3 and therapies involving encapsulation of decellularized tendon scaffolds with cell gels, such as CDSCs or BMSCs, which exhibit potent differentiation abilities for osteogenesis and tenogenesis. Moreover, exosomes derived from stem cells and platelet‐rich plasma (PRP) combined with other agents have demonstrated potential in accelerating healing and modulating inflammatory responses at the tendon‐bone interface. Other approaches, such as miRNA manipulation and lncRNA regulation, hold promise in promoting osteogenic differentiation and tissue regeneration. These findings underscore the diverse strategies being investigated to enhance tendon‐bone healing outcomes, with implications for improving clinical treatments and patient outcomes.

The transforming growth factor‐beta (TGF‐β)/SmadSmad signaling pathway acts as a pivotal regulator of cell growth, differentiation, and migration.^106^ It governs the expression of genes involved in chondrogenic and osteogenic development, such as SRY (sex‐determining region Y)‐box 9 (Sox9), Runx2, and Osterix.^107^ Recent studies underscore the essential role of TGF‐β/Smad signaling in differentiation of TDSCs. Activation of this pathway initiates the phosphorylation of intracellular Smad proteins, which translocate to the nucleus and control the expression of chondrogenic genes, like SOX9, aggrecan, and collagen type II.^108^, ^109^ Additionally, TGF‐β/Smad signaling influences osteogenic differentiation in a context‐dependent manner. Beyond Smad‐dependent pathways, TGF‐β can activate Smad‐independent pathways like MAPK/ERK (extracellular signal‐regulated kinase), PI3K/Akt (phosphoinositide 3‐kinase/protein kinase B), and RhoA/ROCK(rhoA/rho‐associated protein kinase), which further modulate chondrogenic and osteogenic differentiation.^106^, ^107^, ^109^, ^110^

Bone morphogenetic proteins (BMPs), part of the TGF‐β superfamily, play pivotal roles in bone and cartilage development. They activate the Smad1/Smad5/Smad8 pathway, distinct from TGF‐β/Smad2/Smad3, influencing osteogenic and chondrogenic genes.^107^, ^110^ Despite their name, certain BMPs, like BMP3 and BMP13, exhibit inhibitory effects on bone formation. In contrast, BMP2, BMP4, BMP6, BMP7, and BMP9 are recognized osteogenic BMPs that induce bone formation. These pathways' integration within TDSCs differentiation highlights their complex interactions.^111^ Bone morphogenetic proteins (BMP2, BMP7) play a specific role in bone remodeling, resulting in osseointegration.^73^, ^96^, ^112^

Acellular matrices sourced from dermal and bone origins have gained traction for clinical tendon repair. However, the distinctive biological and physical attributes of matrices, contingent upon their tissue sources, may sway the trajectory of cell differentiation.^113^ Connective tissue growth factor (CTGF) and ascorbic acid treatment of TDSCs to engineer cell sheets instigates heightened tendon formation, while preserving robust expression of chondrogenic genes in TDSCs.^24^ The auspicious potential of biomaterials like bioactive glass and hydroxyapatite, acknowledged for their bone conductivity and integration prowess, in fostering osteogenic differentiation of TDSCs warrants attention. Although the anticipated role of strontium‐substituted hydroxyapatite in graft integration is yet to be substantiated in TDSCs‐mediated tendon‐bone healing.^104^, ^114^

Rigid hydrogels restrain differentiation of TDSCs into tendon, cartilage, and osteogenic lineages, evidenced by the dwindling expression of markers such as thrombospondin 4 (THBS4), tenomodulin (TNMD), scleraxis (SCX), COL2, Runx2, osterix and ALP. Intriguingly, the modulation of matrix stiffness, possibly via focal adhesion kinase (FAK) or ERK1/2 activation, emerges as a pivotal regulator of proliferation and differentiation of TDSCs.^115^ The mitogen‐activated protein kinase (MAPK) pathways, including ERK, c‐Jun N‐terminal kinase, and p38, regulate processes like proliferation and differentiation.^116^ Tendon microdamage can trigger chondrogenic differentiation of TDSCs through endoplasmic reticulum stress and activating transcription factor 4 (ATF‐4)/SOX9 activation. Further research is needed to understand how these pathways control differentiation of TDSCs for potential tissue engineering approaches.^117^

In the context of TDSCs, there is little research on the function of the Hh pathway. But a recent study by Feng *et al*. demonstrated that SUFU deficiency caused enhanced chondrogenic and osteogenic differentiation of Ctsk‐Cre‐expressing tendon‐derived cells *via* upregulation of Hedgehog (Hh) signaling.^118^ The Hedgehog (Hh) signal conspicuously influences wound‐induced tendon ossification and ROS production in osteogenesis of TDSCs through antioxidant pathways, advocating Gli antagonist 58 (GANT58) as a potential remedy. Hypoxic culture promotes osteogenic differentiation of r‐TDSCs *via* ERK1/2 signaling.^119^

Wnt/β‐catenin signaling represses the expression of tendon genes Scx, Mkx, and Tnmd in rat tendon‐derived cells, alongside tempering Smad2 and Smad3 activity. Wnt signaling assumes a pivotal role in pathological calcification, as evidenced by Wnt3a's stimulation of ALP activity, calcium nodule formation, and osteogenic marker expression in TDSCs.^120^ The potential role of the Wnt/β‐catenin pathway in regulating the aging of TDSCs has also been suggested.^121^

Collectively, the fusion of biomaterials and TDSCs offers a promising trajectory for advancing tendon‐bone healing. These biomaterials intricately intersect with signaling pathways encompassing TGF‐β, BMP, MAPK, Wnt/β‐catenin, and Hedgehog, thereby shaping osteogenesis and cartilage formation of TDSCs. Meticulous research remains pivotal in deciphering the nuanced molecular mechanisms underpinning the pathway regulation by these materials, ultimately propelling innovative treatment strategies for musculoskeletal ailments.

## Tendon‐bone Healing and Osteo‐chondrogenic Differentiation in Single‐cell Sequencing

Single‐cell sequencing has become a potent tool by offering thorough insights into cellular heterogeneity and gene expression patterns. This approach identifies various cell types, including fibroblasts, tenocytes, smooth muscle cells, endothelial cells, macrophages, and plasma cells, involved in healing processes.^122^, ^123^ Through the analysis of cell transcriptomes, researchers have identified particular gene expression patterns linked to different stages of cell differentiation. This aids in understanding stem cell heterogeneity and their roles in osteogenesis.

Distinguishing general cell populations from specific subpopulations is essential. For example, the identification of Gli1+ cell subpopulation expressing genes related to cartilage and bone formation is crucial for building the tendon enthesis, which supports force transfer and joint motion.^122^ This delineation of heterogeneity within cell populations and the identification of particular marker genes enable researchers to infer cell communication networks and gain important insights into cellular dynamics and interactions contributing to tendon–bone healing, thus informing targeted therapeutic strategies.

Single‐cell RNA sequencing has led to the discovery of a chondrocyte‐like osteoprogenitor (COP) among Gli1+ metaphyseal mesenchymal progenitors, which are a main source for osteoblasts in postnatal mice.^124^ COP, which needs Hh and IGF signaling for bone formation, has a higher potential for chondrogenic and osteogenic differentiation. This finding enhances our understanding of the heterogeneity within stem cell populations and its relevance to tendon–bone healing, suggesting that targeting this subpopulation of cells may enhance the healing process.

Single‐cell sequencing plays a pivotal role in investigating the bone microenvironment and its effect on stem cell osteogenic differentiation. For example, single‐cell sequencing was used to characterize the cellular composition of human femoral head histiocytes and identify prss23 and mxra8 as novel bone metabolism related genes.^125^ The identification of Zinc finger protein 36, C3H like 1 (ZFP36L1), and defense α 3 (DEFA3) as novel genes connected to bone metabolism underlines their potential significance. Additionally, the involvement of Resistin‐capping protein complex 1 (RETN‐CAP1) in the interaction between osteoclasts and immune cells emphasizes the crucial role of the bone immune microenvironment in osteoporosis or osteopenia pathogenesis. Under inflammatory conditions, macrophages are encouraged to multiply and secrete cytokines, thereby inducing the development of osteoclasts.^126^ The knowledge gained from single‐cell sequencing studies holds immense potential for advancing regenerative medicine and developing targeted therapies for bone and tendon disorders. By delineating cell subpopulations, identifying regulators, and revealing novel genes and pathways, single‐cell sequencing enables tailored therapeutic strategies for improved bone and tendon healing.

## Clinical Application and Frontier Technology Prospect of Tendon‐derived Stem Cells

**FIGURE 4:**
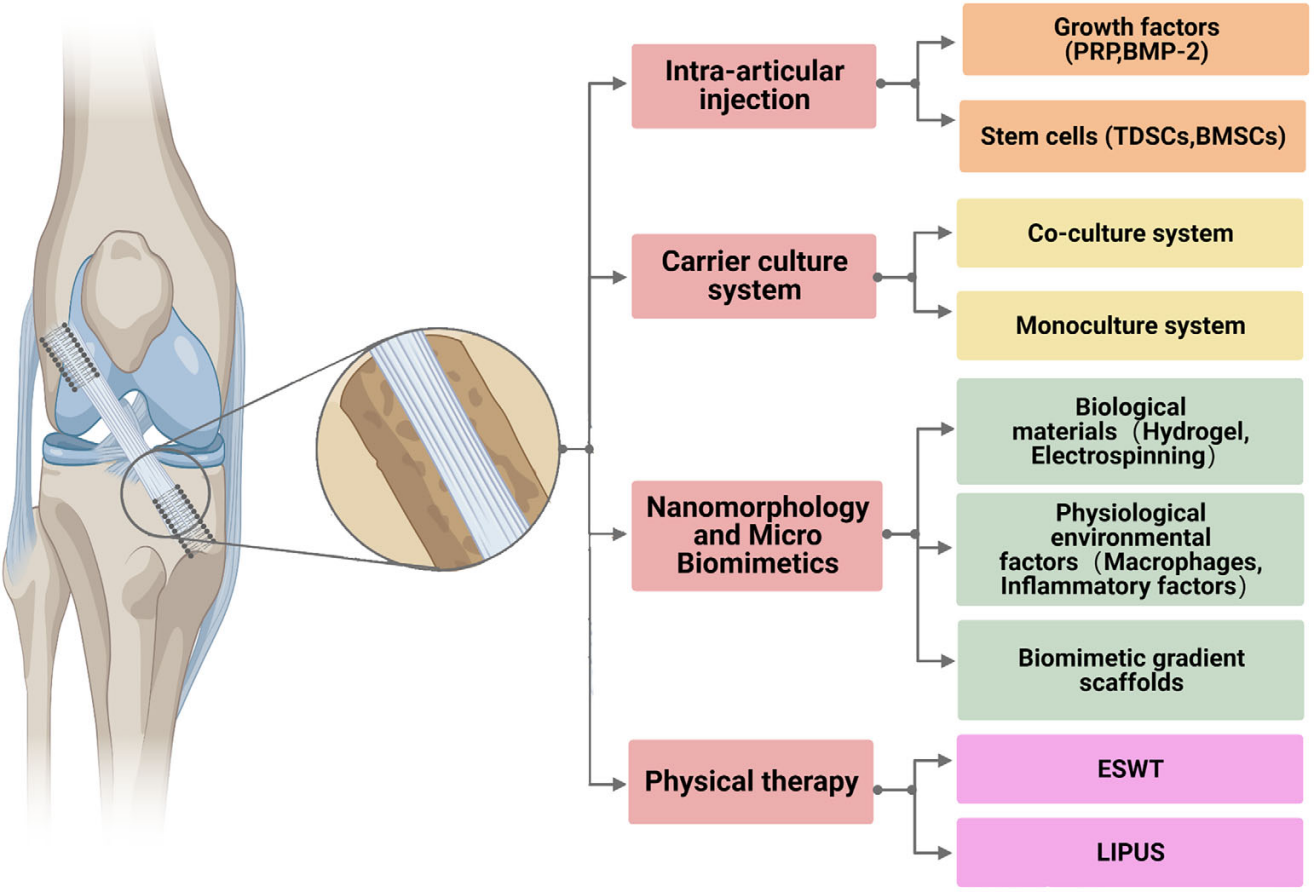
Schematic diagram of strategies for tendon‐bone healing after ACLR. PRP, platelet rich plasma; BMP‐2, bone morphogenetic protein‐2; TDSCs, tendon derived stem cells; BMSCs, bone mesenchymal stem cells; LIPUS, low intensity pulsed ultrasound; ESW, extracorporeal shock wave. Created with BioRender.com.

TDSCs have demonstrated their potential in promoting tendon–bone healing both *in vitro*, through enhanced cell differentiation and extracellular matrix synthesis, and in small animal models, by improving tissue regeneration and biomechanical properties (Figure 4).We also need to pay attention to the current or upcoming clinical strategies for TDSCs, focusing on two types: intra‐articular injection of growth factors and stem cells and carrier culture systems.

Intra‐articular injection represents a minimally invasive method for injecting stem cells, growth factors, and other substances into the joint space, encouraging tendon–bone repair. And they have advantages of minimal invasion and easy application.^127^ One example is the use of platelet‐rich plasma (PRP) injections for the treatment of RC injuries. PRP contains various growth factors that enhance the healing process.^128^ However, the retention and survival of implanted cells at the tendon–bone junction remain questionable. An appropriate carrier or adhesion system is needed to enhance cell localization and function at the target site. The method has several limitations, including unpredictable effects, challenges with dosing and administration, and possible immune reactions or infections.

Carrier culture systems provide a platform for improving TDSCs' performance and encouraging tendon–bone repair. Several studies have explored the use of these cell types and their interactions in tissue engineering approaches for tendon–bone healing. For instance, a study by Li *et al*. showed the effectiveness of using bone marrow‐derived MSCs and TDSCs in combination with a scaffold for improving tendon–bone repair.^9^ However, precise control of the ratio and position of multiple cell types, such as osteoblasts and chondrocytes, on the same carrier presents challenges.^19^ One study employed a co‐culture system of osteoblasts and osteoclasts on BTE scaffold, demonstrating that co‐culture modeling of bone microenvironment provides reliable information on bone cell crosstalk which can promote bone remodeling.^129^ Monoculture includes growing TDSCs alone on a carrier, controlling differentiation by adding particular growth factors or transcription factors. The advantage lies in maintaining the purity and specificity of TDSCs. But replicating complex physiological circumstances is challenging, potentially resulting in reduction of cell activity or proliferative capacity. A study by Yin *et al*. demonstrated the potential of nano‐topography to induce osteogenic differentiation of TDSCs *in vitro*, with randomly oriented fibrous scaffold induced osteogenesis, while the aligned scaffold hindered the process.^130^ Understanding their specific roles and interactions is the key for creating successful tissue engineering solutions for tendon–bone repair.

Moreover, strides in nano‐morphology and micro biomimetic strategies have propelled the development of novel biomaterials and scaffolds. These nanostructured materials, inspired by the natural tendon extracellular matrix, have the capacity to influence TDSCs behavior positively.^131^ Combining physiological environmental factors with biomimetic gradient scaffolds provides an ideal milieu to promote TDSCs proliferation, migration, and differentiation, ultimately enhancing tendon‐bone healing. In conjunction with these tissue engineering approaches, physical therapy modalities like extracorporeal shock wave therapy (ESWT) and low‐intensity pulsed ultrasound (LIPUS) have shown potential in tendon‐bone healing, stimulating collagen synthesis, and promoting tissue regeneration.^39^ The clinical potentials of TDSCs are promising if major obstacles are successfully surmounted.

## Conclusion

Numerous studies have been conducted on tendon‐bone healing, and new tissue engineering strategies offer promising potential for enhancing tendon–bone healing following ACL and RC reconstruction. The application of TDSCs is highly alluring, as they have significant proliferation and differentiation capacities, essential for rebuilding the enthesis. *In vitro* studies have identified culture conditions that can effectively induce osteogenic, and chondrogenic differentiation of TDSCs, thereby holding potential for clinical application. Although TDSCs share some markers with BMSCs, but their distinct expression profile suggests unique properties. Important elements and signaling pathways that can be targeted to control TDSCs differentiation include MAPK, TGF‐β, BMP, Wnt, and Hedgehog. Single‐cell sequencing provides a novel and distinctive perspective on TDSCs and contributes to our understanding of their behavior. Transplantation of cultured TDSCs, alone or in combination with bio‐scaffolds and/or growth factors, shows promise in promoting tendon–bone repair. It is essential to develop approaches that facilitate regeneration of all enthesis zones, including fibrocartilage, calcified cartilage, and bone compartments. Integration of endogenous and exogenous methods, such as cell therapy combined with tailored microenvironments, along with fine‐tuning the spatiotemporal delivery of regulatory signals, represents exciting avenues for future research aiming to achieve functional enthesis regeneration. Further investigations are warranted to elucidate the signaling pathways and mechanisms governing TDSCs differentiation from tendon sources to devise improved tendon‐bone healing strategies for clinical translation.

## Conflict of Interest Statement

This work is supported by The National Natural Science Foundation of China (No. 82202776; No. 82072427; No. 82272557). The authors declare no other conflicts of interest.

## Ethical Statement

We certify that this manuscript is original and has not been published elsewhere, and that the study has not been split up into several parts to increase the quantity of submissions and submitted to various journals or to one journal over time. No data have been fabricated or manipulated (including images) to support the conclusions. No data, text, or theories by others are presented as if they were our own. The submission has been received explicitly from all co‐authors. And authors whose names appear on the submission have contributed sufficiently to the scientific work and therefore share collective responsibility and accountability for the results.

## Author Contributions

All authors have full access to the data in the study and take responsibility for the integrity of the data and the accuracy of the data analysis. Sinuo Shen and Yucheng Lin have equal contribution to this work, they work together to complete the design and writing. Conceptualization, Sinuo Shen and Yucheng Lin. Writing—original draft, Sinuo Shen. Writing—review and editing, Sinuo Shen, Yucheng Lin and Jiachen Sun. Investigation, Yuanhao Liu and Yuzhi Chen. Supervision, Jun Lu. Project Administration, Jun Lu.

## Funding Information

This work is supported by The National Natural Science Foundation of China (No. 82202776; No. 82072427; No. 82272557).
